# Neural maturation enhanced by exercise-induced extracellular derivatives

**DOI:** 10.1038/s41598-020-60930-6

**Published:** 2020-03-03

**Authors:** Hyo Youl Moon, Kyeong Jin Yoon, Won Sang Lee, Hae-Sung Cho, Do-Yeon Kim, Ji-Seok Kim

**Affiliations:** 1 Department of Physical Education, Seoul National University, Seoul, Korea; 2 Institute of Sport Science, Seoul National University, Gwanak-ro, Gwanak-gu, Seoul, 08826 Republic of Korea; 3 School of Biological Sciences, Seoul National University, Seoul, 08826 Korea; 4 Department of Pharmacology, School of Dentistry, Brain Science and Engineering Institute, Kyungpook National University, Daegu, 41940 Republic of Korea; 5 Department of Physical Education, Gyeongsang National University, Jinju-daero, Jinju, 52828 Republic of Korea

**Keywords:** Neurophysiology, Molecular medicine

## Abstract

Physical activity has profound effects on neuronal progenitor cell growth, differentiation, and integration, but the mechanism for these effects is still ambiguous. Using a mouse model, we investigated the effects of two weeks of treadmill running on the dynamics of the size distribution and miRNA profiles of serum extracellular derivatives (EDs) using particle-sizing analysis and small RNA sequencing. We found that an increased average diameter of EDs in the running group compared with the sedentary group (p < 0.05), and 16 miRNAs were significantly altered (p < 0.05) in the running group. Furthermore, functional annotation analysis of differentially expressed miRNA-predicted target genes showed that many of these target genes are involved in the PI3K-Akt pathway. Exercise-induced serum EDs increased Neuro2A cell viability and Akt phosphorylation. We also found that expression levels of neuronal maturation markers such as Microtubule-Associated Protein 2 (MAP2ab) and Neuronal nuclei (NeuN) were increased (p < 0.05, respectively), and that inhibition of the PI3K-Akt pathway by LY294002 pre-treatment ameliorated their expression in Neuro2A cells. Finally, the administration of exercise-induced EDs for 3 days increased the Histone 3 phosphorylation and β-III tubulin expression in Ink/Arf null neural stem cells and progenitors (NSPCs) under each proliferation and differentiation condition. These results suggest that exercise-induced circulating EDs may mediate neuronal maturation during exercise.

## Introduction

Enhanced synaptic plasticity, neurogenesis and neuronal maturation by prolonged exercise mediate improved aspects of brain function, such as motor skills, cognition and mood^1,2^. Neuronal maturation is a continuous developmental process that modifies shape, structure and functional adaptations, playing a pivotal role in the augmentation of the neuronal network^3^. Evidence suggests that the neurotrophic signaling system, neurotransmitter system, inflammatory system and cerebral vascular system are involved in neuronal maturation-related brain function and behaviors^4–8^.

Different types of physical activity have been shown to influence neuronal activities such as neurogenesis and maturation in various regions of the brain^9–11^. Aerobic exercise increases cerebral blood flow (CBF) and angiogenesis in the motor cortex, which is linked to neurogenesis in mice and humans^11,12^. Running also plays a role in neuronal maturation in mice^13^. However, the effects of resistance exercise on neurogenesis remain controversial. Miriam *et al*. found that resistance exercise does not affect adult hippocampal neurogenesis in rats, while Nagamatsu *et al*. found that resistance exercise improves cognitive and functional brain plasticity in the elderly^14,15^. The underlying mechanism for the beneficial effects of physical activity on the brain is currently being investigated. Research has focused on factors such as IRISIN, APLN (Apelin), CTSB (Cathepsin B) and many interleukins that circulate during exercise^16–20^. The exercise-mimetic effects of each factor have been investigated based on behavioral studies using chemical injection or transgenic mice^21^. These studies suggest that various factors mediate cell-to-cell communication during exercise to achieve the observed effects.

Extracellular derivatives (EDs) includes various peptide, lipoproteins, albumin and extracellular vesicles (EVs) that have lipid bilayer structures enclosing small RNA, mitochondrial DNA, and peptide molecules that are released by cells^22,23^. Interestingly, some myokines induced by exercise are found in EV databases such as Vesiclepedia and ExoCarta^22^. These vesicles play crucial roles in intercellular communication in multiple biological processes^24,25^. EVs are classified as exosomes, microvesicles (MVs) or apoptotic bodies based on their size, function and various makers^24^. Research on exercise-induced EVs suggests that exosomes may play a role in mediating the beneficial effect of exercise on metabolic diseases^22,26^. However, the underlying mechanism for how exercise-induced EVs regulate cellular processes is not yet clearly understood, particularly with respect to neuronal function. Because of the purification issues^27^, we expanded our research into EDs containing lipoproteins and EVs. In the present study, we investigated the role and pathway of exercise-induced EDs on neuronal cell viability and maturation.

## Materials and Methods

### Animal study

For the animal experiments, male C57/BL6 (B6) mice (6 weeks) were purchased from Central Laboratory Animal Inc. (Seoul, Korea). Mice were habituated in an animal facility at least for 3 days. A total of 20 mice were randomly assigned to two groups and housed in standard conditions with food and water ad libitum. Mice in the exercise group (n = 10) were acclimated to moderate treadmill running (8 m/min) for 2 days. Treadmill exercise was performed using previous protocol^28^ with little modification. They were then subjected to 2 weeks of exercise training (30 min, twice per day with 1-hour rest, 5 days/week). The mice were trained on a treadmill with progressive increases in intensity. At the end of 2 weeks, all exercise-trained mice were running at a speed of 16 m/min. The day after the last workout, half of the groups (n = 5) were performed the behavioral test and the other half (n = 5) were sacrificed for the blood collection through cardiac puncture. Serum was obtained from the collected blood in heparin-treated tubes with 10 minutes of centrifugation at 2000 g. The animal-handling procedures were based on National Institutes of Health guidelines for animal studies. The experimental procedure was approved by the animal ethical review board of Seoul National University (IACUC-181205-3-1).

### EDs isolation and purification

For the EDs isolation, nomenclature and functional characterization, we tried to follow the minimal information for studies of extracellular vesicles 2018 (MISEV2018) guidelines^29^. However, we still have issues in removing various kinds of lipoproteins^27^ and albumin, so we used extracellular derivatives (EDs) instead of using extracellular vesicles EVs. Total EDs were isolated and purified from 200 mL of serum using the miRCURY Kit (Cat. 76603, Qiagen, CA) according to the manufacturer’s instructions, with minor modification. Briefly, using 0.22 μm syringe filters (Millipore, MA), serum samples were filtered to avoid larger particles. Pre-filtered samples were then mixed with Precipitation Buffer A for 12 hours and centrifuged at 1500 × *g* for 30 minutes. The precipitated EDs were eluted with filtered 1x PBS (100 μl) and used for further analysis. The remaining samples were stored at −20 °C for less than 10 days.

### EDs size analysis

The mean particle diameter-size and size distribution of the EDs were measured by dynamic light scattering (DLS), using a zeta potential and particle size analyzer (ELSZ-1000, Otsuka Electronics, Osaka, Japan) at fixed detector angles of 90° and 25 °C^30^. Dispersed samples in 2 ml of PBS (pH 6.5, dilution factor; 1:20) were then transferred to a transparent cuvette and inserted into the ELSZ-1000 instrument. The particle size was expressed as mean diameter (Z-average) and the particle size distribution was expressed in terms of intensity (differential and cumulative, %).

### Neuro2A cell culture

Neuro2A cells (ATCC CCL-131, USA), a mouse neuroblastoma cell line, were seeded into 6-well plates at an initial density of 10^4^ cells/well and grown in Eagle’s Minimum Essential Medium (EMEM; Gibco, NY, USA) supplemented with exosome-depleted fetal bovine serum (FBS, Gibco) to a final concentration of 10% in a 37 °C incubator with 95% air and 5% carbon dioxide (CO_2_). The cells were passaged every second day and confluency was maintained below 90%. For differentiation of Neuro2a cells, we reduced FBS to 0.5%.

### Neural stem/progenitor cell (NSPC) culture and differentiation induction

The immortalized Ink/Arf−/− NSPCs^31^ harboring multi-lineage differentiation capability were maintained in N2 media supplemented with 20 ng/ml EGF (epidermal growth factor) and bFGF (basic fibroblast growth factor). For differentiation induction, NSPCs were dissociated into single cells using TrypLE (Life Technologies), and plated on polyornithine and fibronectin-coated plates in N2 culture medium including 1% fetal bovine serum (FBS) and B27 Supplements (Life Technologies) without growth factors for 3 days.

### Cellular viability assay

Cell viability was quantified using Cell Counting Kit-8 (CCK-8; Dojin Laboratories, Kumamoto, Japan). Briefly, Neuro2A neuroblastoma cells were seeded into 96-well plates at an initial density of 10^3^ cells/well. After 48 hours, the medium was changed to serum-free EMEM. After incubation with the vehicle (phosphate-buffered saline; PBS) or the indicated concentrations (1, 10, and 100 ng/μl) of EDs from the sedentary or exercise group for the 24 hours, then reagent was added for 3 more hours. 10% FBS was used as positive control. To assess cell viability, absorbance at 450 nm was measured using a Tecan (Infinite 200 PRO series) 96-well microplate spectrophotometer (Mannedorf, Switzerland).

### Western blot analysis

Neuro2A cells were harvested using RIPA (radioimmunoprecipitation) buffer (Millipore), Protease/Phosphatase Inhibitor Cocktail (Cell Signaling Technology, Danvers, MA) and sample buffer (Bio-Rad). For Western Blot analysis, 20 μg of protein was used for 4–12% sodium dodecyl sulfate polyacrylamide gel electrophoresis (SDS-PAGE) and transferred to an NC (nitrocellulose) membrane using a semi-dry kit (Invitrogen). The membranes were blocked for 30 minutes with 5% nonfat dried milk in Tris-buffered saline (TBS) containing 0.1% Triton X-100. Primary antibodies against CD9, AKT, P-AKT (S473), and β-Tubulin were purchased from Cell Signaling Technology (Beverly, MA, USA), used at a dilution of 1:1,000, and incubated at 4 °C overnight. Secondary anti-rabbit and anti-mouse antibodies were used at a dilution of 1:5,000. Specific signals were visualized using LAS500 (GE Healthcare Life Sciences).

NSPCs were disrupted directly with laemmli buffer (60 mM Tris-HCl (pH 6.8), 2% (w/v) SDS, 10% (v/v) glycerol, 0.02% (w/v) bromophenol blue), followed by sonication and heat-denaturation at 95 °C. Samples were fractionated by SDS-PAGE and transferred to a PVDF (Polyvinylidene fluoride or polyvinylidene difluoride) membrane. After blocking membranes with 5% non-fat dried milk in TBST (10 mM Tris, pH 8.0, 150 mM NaCl, 0.5% Tween 20) for 30 min, the membrane was washed with TBST and incubated with antibodies against β-III tubulin (1:1,000, Abcam), phosphor Histone3 at Ser 10 (1:1,000, Cell Signaling), β-Actin (1:5,000, Sigma Aldrich), total Histone3 (1:10,000, Cell Signaling) overnight at 4 °C. Next day, membranes were washed three times (10 min each) with TBST and incubated with horseradish peroxidase-conjugated anti-mouse (1:10,000, Bethyl Laboratories) or anti-rabbit antibodies (1:5,000, Bethyl Laboratories) for 1 hour. Membranes were washed with TBST and signals were detected with D-PlusTM ECL Femto system (Dongin LS). Quantification of Western Blots were performed with ImageJ.

### miRNA and target gene expression analysis

The quality of the raw data was checked in a pre-processing step, and adapter sequences existing in small RNA-seq reads were removed. The results were generated by mapping to annotated miRNA sequences using pre-processed readings. For differential miRNA expression analysis, the R package was used to normalize the expression and variance to reduce the differences among the samples. Using DESeq 2, we constructed a statistical model with a negative binomial as a reference distribution by using raw read count as the input and analyzed differences in expression. Functional analysis (gene set enrichment analysis) and DEG were performed using the obtained P-value and fold change. For the selection of predicted target genes, databases from miRanda, miRBase, TargetScan, microT, PicTar were used.

### PCR array analysis

Total RNA was extracted from Neuro2A neuroblastoma cells using a total RNA extraction kit (Ribozol, Amresco) following the manufacturer’s manual. Then, 5 μg/10 μl RNA was used for cDNA synthesis. First-strand cDNA was synthesized by RT using oligo (dT) primers and SuperScript II reverse transcriptase (Invitrogen). For PCR array, total RNA (100 ng) was amplified using the SYBR Green Master Mix (Thermo Fisher) and analyzed using CFX96 qPCR instruments (Bio-Rad). The primers used in the PCR reaction were as follows: MAP2ab; f(forward) 5′-TTGGTGCCGAGTGAGAAGA-3′ r(reverse) 5′-GTCTGGCAGTGGTTGGTTAA-3′, NeuN; f 5′-CCAAGCGGCTACACGTCT-3′ r 5′-GCTCGGTCAGCATCTGAG-3′, Tbp; f 5′-ACCCTTCACCAATGACTCCTATG-3′ r 5′-ATGATGACTGCAGCAAATCGC-3′.

### Data analysis

Statistical analyses were carried out using Prism 5.0. Comparisons between groups were performed with one-way analysis of variance (ANOVA) for the Western Blot, Real-time PCR and CCK-8 assays, while Student’s *t*-test was used for all other analyses. All values are expressed as mean ± standard error of the mean (SEM).

## Results

### Effects of 2 weeks of treadmill on mice grip strength and motor function

**Figure 1:**
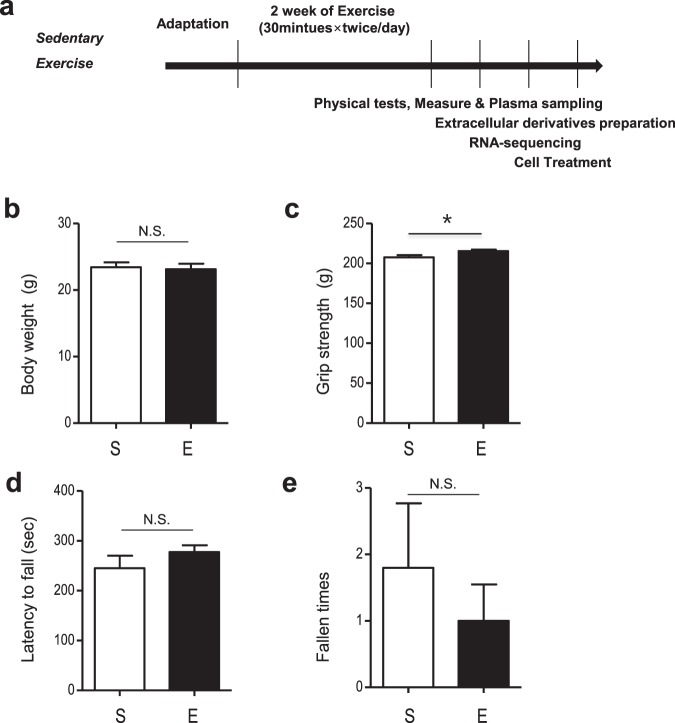
Schematic design of experiments and physical and behavioral changes after 2 weeks of exercise. (**a**) Experimental design for 2 weeks of treadmill exercise. (**b**) Comparison of body weights in the sedentary and exercise group after 2 weeks. (**c**) Grip strength comparison between the sedentary and exercise group. (**d**,**e**) Latency to fall and fall times in the rotarod test. The data represent means ± SEM (*p < 0.05, n = 5).

To study the effect of running on serum EDs, an inclining speed treadmill protocol was applied to mice twice per day for two weeks (Fig. 1a). Treadmill running had no effect on body weight (p > 0.05, Fig. 1b). However, grip strength increased after two weeks of treadmill training (p < 0.05, Fig. 1c). Motor function was assessed using a 5-minute rotarod performance test. There was no difference between the groups in latency to first fall or fall time (p > 0.05; Fig. 1d,e).

### Characterization of exercise-induced extracellular derivativies(EDs): Protein concentration & size analysis

**Figure 2:**
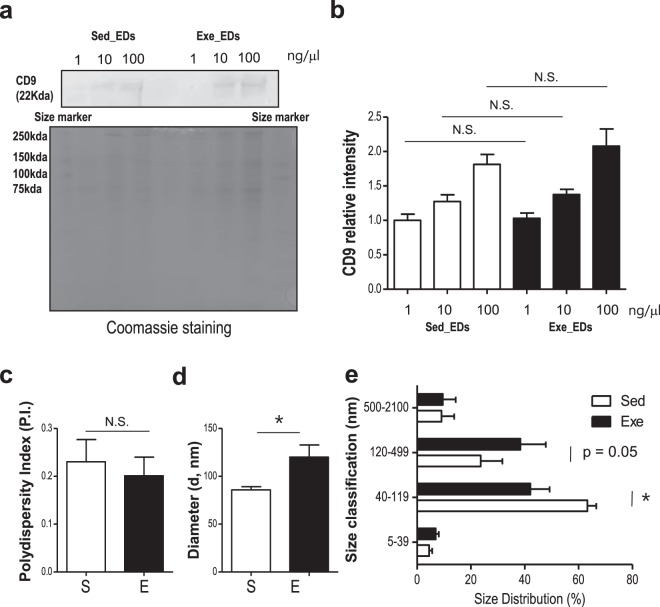
Characterization of the EDs isolated from mice in the running group and sedentary group (**a**,**b**) WB analysis of CD9, an exosome marker, in EDs from mice in the running group and sedentary group (**b**) Relative CD9 intensity. Coomasie staining was used as a loading control. (**c**) There was no difference between groups in the diversity of EDs. (**d**) Average diameter was significantly larger in the EDs from mice in the running group compared with those in the sedentary group. (**e**) Size distribution data shows that exercise decreased the proportion of EDs 40–119 nm, while increased the proportion of EDs 120–499 nm. EDs, Exercise derivatives, S or Sed, sedentary, E or Exe, exercise. The data represent means ± SEM (*p < 0.05, N.S., not significant).

RNA and protein concentration were detected by Nano-100 (Allsheng, China). No significant alterations were found in EDs from mice in the 2 weeks of treadmill running group vs. the sedentary group (RNA [sed; 0.17 ± 0.07 μg/μl, exe; 0.16 ± 0.07 μg/μl], protein [sed; 13.72 ± 2.43 mg/ml, exe; 12.49 ± 1.45 mg/ml], and p > 0.05, n = 5, respectively). As shown in Fig. 2a,b, no changes were detected (p > 0.05) in the level of CD9 in EDs isolated from the serum of mice in the running group compared with mice in the sedentary group. These results confirmed that EDs contains EVs. Next, the size of EDs was determined by dynamic light scattering (DLS) using particle size analyzer (ELSZ-1000). The effects of exercise on polydispersity, average particle size and size distribution of EDs are displayed in Fig. 2c–e. No changes were detected in the dispersity of samples (p > 0.05, Fig. 2c) indicating that EDs from each group contained a similar heterogeneity of sizes. However, paired *t*-test analysis showed that exercise increased the average particle diameter of EDs (p = 0.02; Fig. 2d). Because EVs in EDs are classified by their size, we further investigated the size distribution of EDs using four different ranges (Fig. 2e). One-way ANOVA analysis followed by post-hoc testing revealed a significant effect of exercise on size distribution (p < 0.05, Fig. 2e). In particular, running increased the number of EDs between 120 nm to 499 nm in size compared with sedentary mice (p = 0.05) while decreased the number of EDs between 40–119 nm (p < 0.05, Fig. 2e).

### microRNA profiles are altered in exercise-induced extracellular derivatives (EDs)

**Figure 3:**
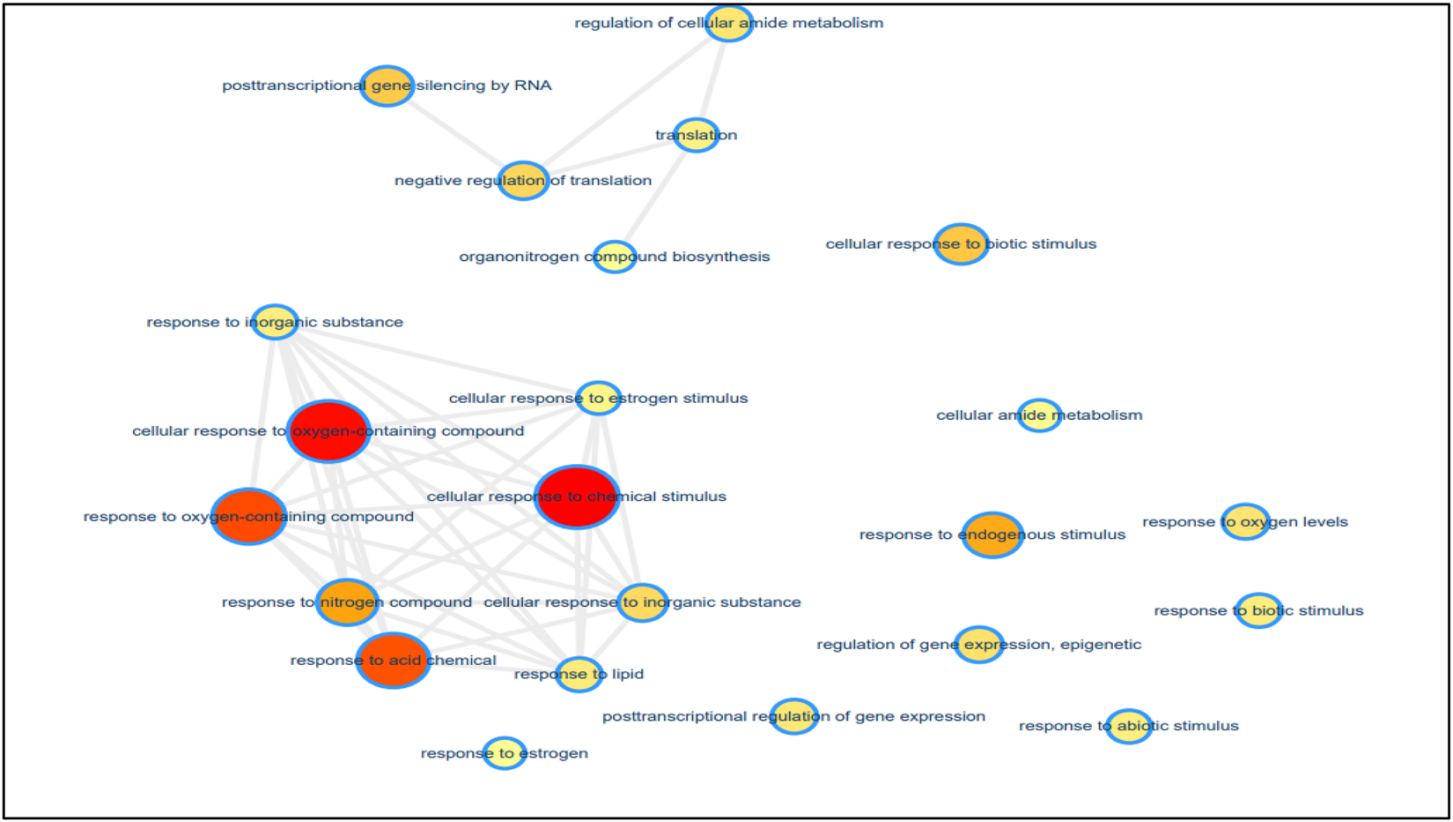
Functional Clustering of miRNAs in EDs. Yellow to red color-scale indicated an increasing number of miRNAs within the group.

Increasing evidence demonstrates that miRNAs are encapsulated in EDs^24,32^. To elucidate the effects of running on miRNA profiles in circulating EDs, we compared the miRNA profiles from serum EVs from mice in the running group and those in the sedentary group. miRNA read counts were normalized with DESeq 2 and significant differences in miRNA expression were assessed. We identified 16 miRNAs, including mmu-miR-101a-3p, -101c,-16-5p, -223-3p, -297a-3p, -297b-3p, -297c-3p, -342-3p, -466f-3p, -466h-3p, -466i-3p, -669c-5p, -674-3p, -676-3p, -706, -7a-5p, and 93-5p, that were significantly increased in EDs from mice in the running group (Table 1). Gene ontology (GO) analysis showed that differentially expressed miRNAs in EDs from mice in the running group are related to the cellular response to chemical stimulus, cellular response to oxygen-containing compound, and cellular response to acid chemical. This indicated that EDs are responsive to the dynamic external environment during exercise (Fig. 3). In addition, highly expressed miRNAs in the EDs from mice in the running group were involved in post-transcriptional regulation and translation. Using miRNA target gene prediction tools, we observed that 193 genes were commonly targeted by the enriched miRNAs in the EDs from mice in the running group. Furthermore, KEGG pathway analysis identified 10 genes (Ddit4, Creb1, Eif4e, Gsk3b, Itgb3, Myb, Rps6kb1, Sgk1, Akt3 and Ywhah) that were involved in the PI3K (phosphatidylinositol 3-kinase) -AKT (Protein Kinase B) signaling pathway (Table 2).

**Table 1:** List of miRNAs.

miRNA	base Mean	log2 Fold Change	p value
mmu-miR-16-5p	455.51	12.59	0.0003
mmu-miR-342-3p	181.08	11.26	0.0046
mmu-miR-466f-3p	212.60	9.04	0.0069
mmu-miR-7a-5p	151.84	11.00	0.0075
mmu-miR-669c-5p	144.99	10.94	0.0085
mmu-miR-676-3p	142.22	−10.28	0.0129
mmu-miR-223-3p	109.52	10.53	0.0176
mmu-miR-297a-3p	107.03	10.50	0.0186
mmu-miR-297b-3p	107.03	10.50	0.0186
mmu-miR-297c-3p	107.03	10.50	0.0186
mmu-miR-466h-3p	86.50	10.19	0.0307
mmu-miR-466i-3p	75.92	10.00	0.0408
mmu-miR-706	1035.61	3.49	0.0412
mmu-miR-674-3p	72.18	9.93	0.0453
mmu-miR-101a-3p	80.35	−9.46	0.0477
mmu-miR-101c	80.35	−9.46	0.0477

**Table 2:** List of miRNA predicted target genes.

Term	No. of Genes
Ubiquitin mediated proteolysis	7
Hippo signaling pathway	7
HTLV-1 infection	9
PI3K-Akt signaling pathway	10
MAPK signaling pathway	7

### Exercise-induced EDs enhance the viability of Neuro2A cells partially via the PI3K-AKT pathway

**Figure 4:**
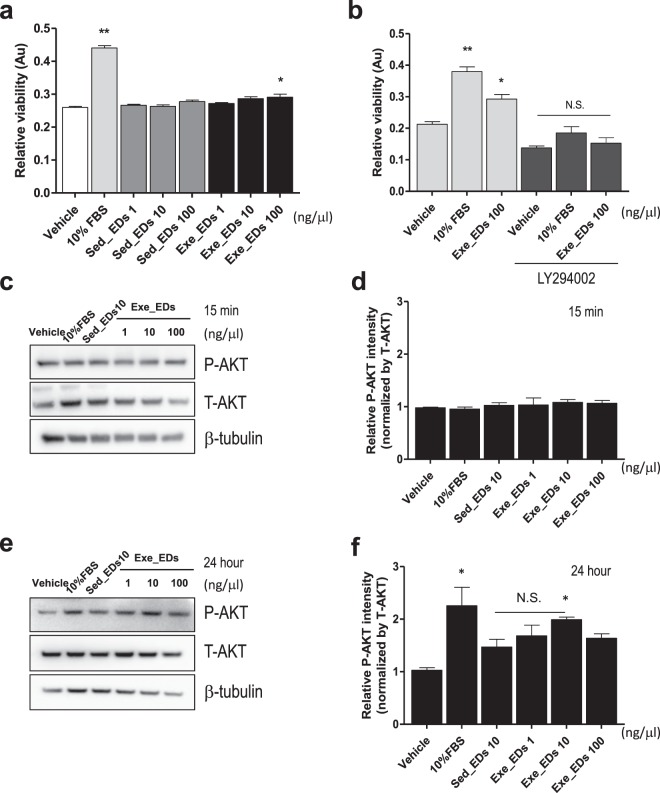
Effect of exercise-induced EDs on viability and AKT phosphorylation in Neuro2A cells. (**a**) 24 hours or treatment with 100 ng/μl EDs from mice in the exercise group increased the viability of Neuro2A cells compared to vehicle (PBS) control or EDs from sedentary mice. (**b**) Pre-treatment with Ly294002 compound for 30 minutes ameliorates the viability of Neuro2A cells induced by exercise-induced EDs. (**c,d**) There was no difference in Akt phosphorylation (S473) after 15 minutes treatment with exercise-induced EDs compared with sedentary EDs or vehicle. (**e**,**f**) 24 hours of 10 and 100 ng/μl treatment with exercise-induced EDs enhanced Akt phosphorylation (S473) (**e,f**) normalized by total-Akt and b-tubulin, respectively. Vehicle; phosphate-buffered saline (PBS), Ly294002; PI3K inhibitor (10 μM). The data represent means ± SEM (**p < 0.01, *p < 0.05, N.S.; not significant).

The PI3K-AKT pathway is a crucial signaling pathway in cellular processes such as proliferation and differentiation^33,34^. We further investigated the effect of EDs from mice in the running group and sedentary group on the viability of neuroblastoma cells. Treating Neuro2A cells with exercise-induced EDs for 24 hours significantly altered their viability compared with sedentary mice EDs or the vehicle-treated control (Fig. 4a). Exercise-induced EDs (100 ng/μl) promoted increased viability of Neuro2A cells compared with the control group (p < 0.05, Fig. 4a). We then used the LY294002 compound, a PI3K-AKT pathway inhibitor, to assess the involvement of the PI3K-AKT pathway in the proliferation of neuroblastoma cells. Pre-treatment with LY294002 compound (10 μM) reduced the viability of Neuro2A cells treated with exercise-induced EDs (p < 0.05, Fig. 4b). We also found that 24 hours of treatment with exercise-induced EDs (at 10 ng/μl) increased AKT phosphorylation (Ser473) in Neuro2A cells compared with the vehicle treatment (p < 0.05, Fig. 4e,f). However, no such effect was observed when EDs were administered for only 15 min (p > 0.05, Fig. 4c,d). The EDs from sedentary mice also tended to increase AKT phosphorylation compared to the control (p = 0.09, Fig. 4f), and this effect was not statistically different from that of exercise-induced EDs (p > 0.05, Fig. 4f).

### Exercise-induced EDs promote neural maturation markers in Neuro2A cells

**Figure 5:**
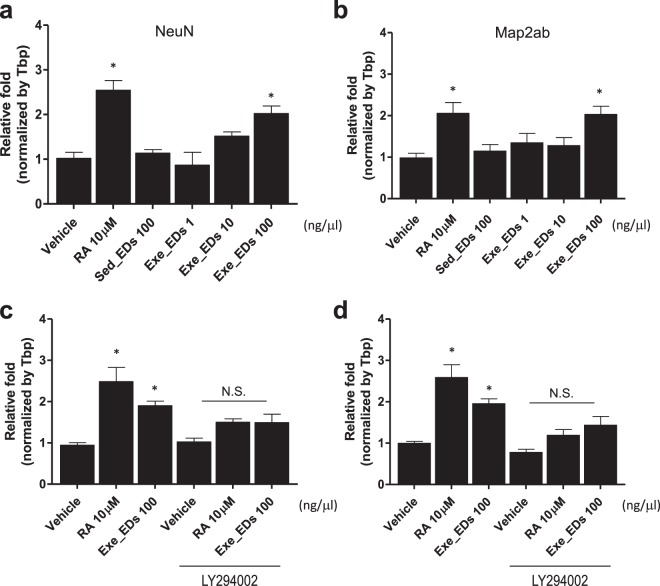
Effect of exercise-induced EDs on genes related to neuronal maturation. (**a**,**b**) mRNA levels of (**a**) NeuN and (**b**) MAP2ab after treatment with EDs from mice in the running group or sedentary group on Neuro2A cells for 24 hours. (**c**,**d**) Pre-treatment with Ly294002 compound for 30 minutes had a significant effect on the levels of (**c**) NeuN and (**d**) MAP2ab mRNA level induced by exercise-induced EDs. Vehicle; phosphate-buffered saline (PBS), RA; retinoic acid (10 μM), Ly294002; PI3K inhibitor (10 μM). The data represent means ± SEM (*p < 0.05, N.S.; not significant).

The AKT pathway plays a crucial role in neuronal cell differentiation via modulation of genes related to synaptic plasticity, such as MAP2ab and NeuN^35,36^. We investigated the effect of exercise-induced EDs on these neuronal maturation markers. The expression levels of both MAP2ab and NeuN (p < 0.05; Fig. 5a,b) were higher in Neuro2A cells treated with 100 ng/μl of exercise-induced EDs for 24 hours compared with cells incubated with EDs from sedentary mice or vehicle (PBS). Subsequent experiments showed that the increased MAP2ab and NeuN expression under exercise-induced EV treatment were ameliorated by LY294002 compound (10 μM) pre-treatment in Neuro2A cells (p > 0.05; Fig. 5c,d).

### Exercise-induced EVs increases the proliferation and differentiation markers in neural stem cells and progenitors (NSPCs)

**Figure 6:**
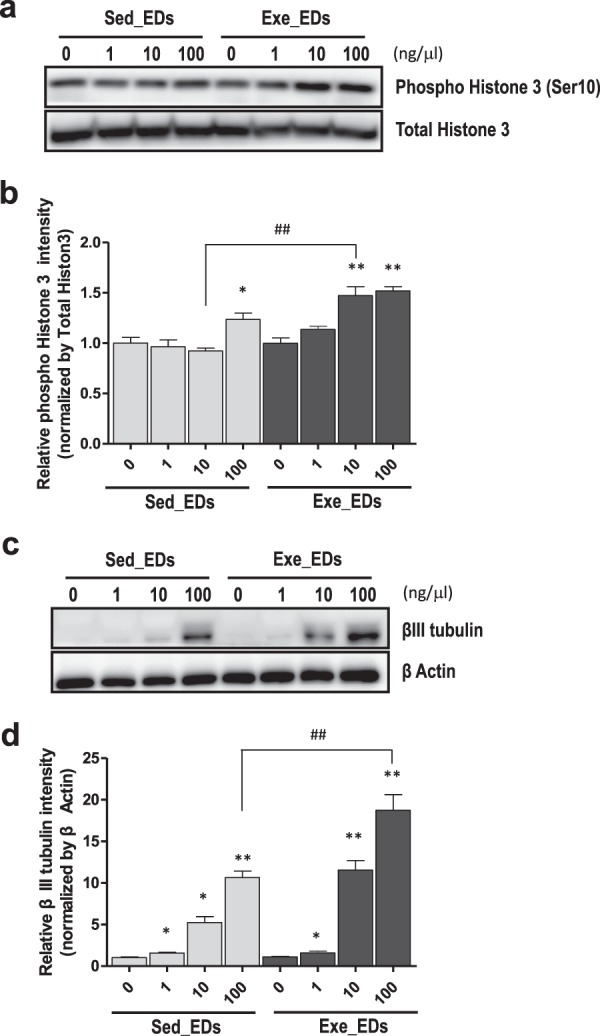
Proliferation marker phosphorylated histone H3 and differntiation marker β-III tubulin are increased by exercise-induced EDs treatment in neural stem cells and progenitors (NSPCs). (**a**) Immunostaining with phosphor - Histone 3 antibody and total Histon 3 after treatment of sedentary-induced EDs or exercise-induced EDs with indicated dosage. (**b**) Quantification of phosphor-histone antibody normalized by total Histon 3. (**c**) Immunostaining with β-III tubulin and β-actin after administration of sedentary-induced EDs or exercise- induced EDs with indicated dosage. (**d**) Quantification of β-III tubulin normalized by total β-actin (*p < 0.05; **p < 0.01; n = 3).

To determine the role of exercise-induced EDs in more physiological condition, we next investigated the effect of the exercise-induced EDs on the expression of proliferation and differentiation markers in NSPCs. Ink/Arf-null NPC (Neural progenitor cell) were treated with 0, 1, 10, 100 ng/μl of exercise-induced EDs or sedentary-induced EDs for 3 days. Phosphor Histone3 (Ser10) was used as a proliferation marker and β-III tubulin was used as a marker for neuronal differentiation. In proliferating conditions, both 100 ng/μl of sedentary-induced EDs and 10, 100 ng/μl of exercise-induced EDs treatment have effect on cell proliferation marker (p < 0.05, p < 0.01, respectively; Fig. 6a,b). In particular, 10 ng/μl of exercise-induced EDs treatment significantly increased the Histone 3 phosphorylation comparing same dosage treatment of sedentary-induced EDs (p < 0.01; Fig. 6a,b). For evaluation of neuronal differentiation, β-III tubulin was used as a marker. When artificially induced differentiation as described in method, both 1, 10, 100 ng/μl of sedentary-induced EDs and exercise-induced EDs treatment have effect on cell differentiation marker (p < 0.05; Fig. 6c,d). Following comparison analysis found that 10 ng/μl of exercise-induced EDs treatment significantly increased the β-III tubulin comparing same dosage treatment of sedentary-induced EDs (p < 0.01; Fig. 6a,b).

## Discussion

Extracellular derivatives (EDs) contains EVs that are small sacs released by cells that contain parent cell-derived materials such as peptides, metabolites, and nucleic acids^24^. Recent studies have identified various physiological roles for EVs in the context of cell excretion^22,26,37^. EVs are known to stimulate or suppress metabolism through intercellular information exchange under various physiological parameters, including exercise^22,24^. Cell-derived hormones and cytokines also play such roles, but EVs are more diverse, target-specific and stable, and are now believed to play a major role as signal transducers that mediate exercise^22,24,32^. Because of these characteristics, recent studies have focused on EVs as signal transporters during exercise^22,38^. However, there are technical issues purifying these EVs, we used extracellular derivatives (EDs) including EVs, albumin and lipoproteins^23,27^. Using a mouse model, we found that treadmill running for 30 minutes twice per day (a total of 1 hour per day) for two weeks increased the diameter of particles in EDs. In particular, the proportion of particles 120–499 nm in diameter increased compared with sedentary mice. Although the process of formation is slightly different from exosomes, microvesicles are also equipped with specific components, including genetic materials^24,32^. Our results indicate that EDs can be actively involved in intercellular communication during exercise.

We characterized the miRNA profiles of the EDs after 2 weeks of treadmill exercise and administered these EDs to neuronal cells to investigate the cellular mechanisms for the neurogenic effects of exercise. miRNAs are small non-coding RNA molecules (about 22 nucleotides) found in various tissues, and have biological functions in cancer, the cardiovascular system, adipogenesis, and neurogenesis, mostly via post-transcriptional regulation of gene expression^39,40^. The majority of miRNAs are located within the cell, but some miRNAs can be secreted from cells and circulate in fluids^39^. These circulating miRNAs have been reported to play roles in biological functions such as atherogenesis, and thrombosis^41,42^. D’Souza *et al*., found circulatory exosomal miRNA following intense exercise was unrelated to muscle and plasma abundance^43^. Furthermore, the presence of miRNAs in EDs has now been reported in several cell types, including mesenchymal stem cells^44^ immune cells^45^, tumor cells^46^ and endothelial cells^47^. We observed that 2 weeks of treadmill exercise altered the profile of miRNAs related to the cellular response to chemical stimulus, cellular response to oxygen-containing compound, and cellular response to acid chemical. The functional categorization of differentially expressed miRNAs sheds light on the dynamics of inter- and intracellular conditions during exercise^48^. We further searched the literature to understand the functions of the altered miRNAs. miR16, -342, -7a, -674, -101, etc. are known to affect neuronal function and have been reported to be involved in energy metabolism^49–56^. miRNAs are known to regulate not only target genes but also their pathways. We identified genes that were predicted to be regulated by at least three miRNAs using a miRNA target gene prediction database (as discussed in the Materials and Methods section). KEGG pathway enrichment analysis was then performed using DAVID with predicted gene-sets. The most significantly enriched pathway was the PI3K-Akt signaling pathway.

The Akt pathway plays a crucial role in survival and growth via modulation of signaling transduction pathways^33,35^. Akt resides in the cytosol in an inactive conformation until the cell is stimulated, then translocates to the plasma membrane^57^. Recent research has focused on the roles of this pathway in the neuronal system. PI3K-Akt signaling is involved in the growth of neuronal stem cells (NSCs) via FOXO phosphorylation and PTEN inhibition^58^. In the present study, we verified that exercise-induced EDs can activate Akt signaling. Long-term treatment with exercise-induced EDs increased Akt phosphorylation and the viability of neuronal cells, although Akt activation was not affected by short-term treatment with exercise-induced EDs. This indicates that EDs require a period of adaptation to act on target cells. It has been reported that overexpression of Akt increases Creb phosphorylation and promotes NSC differentiation^34^. We further investigated the effects of exercise-induced EDs on neuronal maturation markers, including MAP2ab and NeuN. After being incubated with exercise-induced EDs for 24 hours, MAP2ab and NeuN expression increased compared with cells incubated with EDs derived from sedentary mice or vehicle (PBS). To establish whether the Akt pathway was involved in these effects, we conducted further experiments using LY294002, a PI3K inhibitor. Pre-treatment with LY294002 dramatically decreased the expression level of MAP2ab and NeuN in neuroblastoma cells treated with exercise-induced EDs. In accordance with previous studies^59,60^, we found that exercise-induced EDs may be involved in neuronal cell viability and maturation via PI3K-Akt signaling. Consistent with these findings in Neuro2A cells, we further validate the effect of exercise-induced EDs’ on neural stem cell function in NSPCs. Interestingly, we found that the markers involved in the proliferation and differentiation of neural stem cells were affected even in the sedentary-induced EDs, but there was a more significant effect in the exercise-induced EDs. Since the PI3K-Akt pathway plays an important role in glucose metabolism^58^, it should be confirmed whether exercise-induced EDs can contribute to the energy metabolism system during exercise. Further studies are necessary to elucidate the characteristics of functions of exercise-induced EDs in various conditions.

Understanding the underlying mechanisms for exercise-induced neuronal maturation may facilitate the development of evidence-based exercise programs for participants with neurodegenerative diseases. Our findings confirm that exercise-induced EDs can act as a communication element during exercise. In particular, exercise-induced EDs contribute to the proliferation and maturation of neurons, which are related to the beneficial effects of exercise on cognitive function and emotion.

## References

[1] Voss MW (2019). Exercise and Hippocampal Memory Systems. Trends Cogn. Sci..

[2] Cooper C’iana, Moon Hyo Youl, van Praag Henriette (2017). On the Run for Hippocampal Plasticity. Cold Spring Harbor Perspectives in Medicine.

[3] Bassett DS, Sporns O (2017). Network neuroscience. Nat. Neurosci..

[4] Moon HY (2012). Macrophage migration inhibitory factor mediates the antidepressant actions of voluntary exercise. Proc. Natl Acad. Sci. USA.

[5] Johansen-Berg H, Duzel E (2016). Neuroplasticity: Effects of Physical and Cognitive activity on brain structure and function. Neuroimage.

[6] Brichta L (2015). Identification of neurodegenerative factors using translatome-regulatory network analysis. Nat. Neurosci..

[7] Hunsberger JG (2007). Antidepressant actions of the exercise-regulated gene VGF. Nat. Med..

[8] Moon HY, van Praag H (2014). Muscle over mind. Cell Metab..

[9] Cassilhas RC (2007). The impact of resistance exercise on the cognitive function of the elderly. Med. Sci. Sports Exerc..

[10] Gutierrez RMS (2018). Motor improvement requires an increase in presynaptic protein expression and depends on exercise type and age. Exp. Gerontol..

[11] McCloskey DP, Adamo DS, Anderson BJ (2001). Exercise increases metabolic capacity in the motor cortex and striatum, but not in the hippocampus. Brain Res..

[12] van Praag H, Kempermann G, Gage FH (1999). Running increases cell proliferation and neurogenesis in the adult mouse dentate gyrus. Nat. Neurosci..

[13] Zhao C, Teng EM, Summers RG, Ming GL, Gage FH (2006). Distinct morphological stages of dentate granule neuron maturation in the adult mouse hippocampus. J. Neurosci..

[14] Nokia MS (2016). Physical exercise increases adult hippocampal neurogenesis in male rats provided it is aerobic and sustained. J. Physiol..

[15] Nagamatsu LS, Handy TC, Hsu CL, Voss M, Liu-Ambrose T (2012). Resistance training promotes cognitive and functional brain plasticity in seniors with probable mild cognitive impairment. Arch. Intern. Med..

[16] Moon HY (2016). Running-Induced Systemic Cathepsin B Secretion Is Associated with Memory Function. Cell Metab..

[17] Wrann CD (2013). Exercise induces hippocampal BDNF through a PGC-1alpha/FNDC5 pathway. Cell Metab..

[18] Vinel C (2018). The exerkine apelin reverses age-associated sarcopenia. Nat. Med..

[19] Suzuki K (2002). Systemic inflammatory response to exhaustive exercise. Cytokine kinetics. Exerc. Immunol. Rev..

[20] Moon HY (2019). Conditioned media from AICAR-treated skeletal muscle cells increases neuronal differentiation of adult neural progenitor cells. Neuropharmacol..

[21] Guerrieri D, Moon HY, van Praag H (2017). Exercise in a Pill: The Latest on Exercise-Mimetics. Brain Plast..

[22] Safdar A, Saleem A, Tarnopolsky MA (2016). The potential of endurance exercise-derived exosomes to treat metabolic diseases. Nat. Rev. Endocrinol..

[23] Baranyai T (2015). Isolation of Exosomes from Blood Plasma: Qualitative and Quantitative Comparison of Ultracentrifugation and Size Exclusion Chromatography Methods. PLoS One.

[24] Maas SLN, Breakefield XO, Weaver AM (2017). Extracellular Vesicles: Unique Intercellular Delivery Vehicles. Trends Cell Biol..

[25] Schüttler Dominik, Clauss Sebastian, Weckbach Ludwig T., Brunner Stefan (2019). Molecular Mechanisms of Cardiac Remodeling and Regeneration in Physical Exercise. Cells.

[26] Chaturvedi P, Kalani A, Medina I, Familtseva A, Tyagi SC (2015). Cardiosome mediated regulation of MMP9 in diabetic heart: role of mir29b and mir455 in exercise. J. Cell Mol. Med..

[27] Vickers KC, Palmisano BT, Shoucri BM, Shamburek RD, Remaley AT (2011). MicroRNAs are transported in plasma and delivered to recipient cells by high-density lipoproteins. Nat. Cell Biol..

[28] Yoon KJ, Zhang D, Kim SJ, Lee MC, Moon HY (2019). Exercise-induced AMPK activation is involved in delay of skeletal muscle senescence. Biochem. Biophys. Res. Commun..

[29] Thery C (2018). Minimal information for studies of extracellular vesicles 2018 (MISEV2018): a position statement of the International Society for Extracellular Vesicles and update of the MISEV2014 guidelines. J. Extracell. Vesicles.

[30] Park SJ, Garcia CV, Shin GH, Kim JT (2017). Development of nanostructured lipid carriers for the encapsulation and controlled release of vitamin D3. Food Chem..

[31] Hwang I (2018). Far Upstream Element-Binding Protein 1 Regulates LSD1 Alternative Splicing to Promote Terminal Differentiation of Neural Progenitors. Stem Cell Rep..

[32] Trovato E, Di Felice V, Barone R (2019). Extracellular Vesicles: Delivery Vehicles of Myokines. Front. Physiol..

[33] Shioda N, Han F, Fukunaga K (2009). Role of Akt and ERK signaling in the neurogenesis following brain ischemia. Int. Rev. Neurobiol..

[34] Peltier J, O’Neill A, Schaffer DV (2007). PI3K/Akt and CREB regulate adult neural hippocampal progenitor proliferation and differentiation. Dev. Neurobiol..

[35] Wang Y (2017). Primary Blast-Induced Changes in Akt and GSK3beta Phosphorylation in Rat Hippocampus. Front. Neurol..

[36] Shu T (2018). Effects and mechanisms of matrix metalloproteinase2 on neural differentiation of induced pluripotent stem cells. Brain Res..

[37] Aminzadeh MA (2018). Exosome-Mediated Benefits of Cell Therapy in Mouse and Human Models of Duchenne Muscular Dystrophy. Stem Cell Rep..

[38] Li Gaohua, Liu Hua, Ma Chunlian, Chen Yanfang, Wang Jinju, Yang Yi (2019). Exosomes are the novel players involved in the beneficial effects of exercise on type 2 diabetes. Journal of Cellular Physiology.

[39] Gebert LFR, MacRae IJ (2019). Regulation of microRNA function in animals. Nat. Rev. Mol. Cell Biol..

[40] Pons-Espinal M (2019). MiR-135a-5p Is Critical for Exercise-Induced Adult Neurogenesis. Stem Cell Rep..

[41] Mause SF, Weber C (2010). Microparticles: protagonists of a novel communication network for intercellular information exchange. Circ. Res..

[42] Sluijter JP (2010). MicroRNA-1 and -499 regulate differentiation and proliferation in human-derived cardiomyocyte progenitor cells. Arterioscler. Thromb. Vasc. Biol..

[43] D’Souza RF (2018). Circulatory exosomal miRNA following intense exercise is unrelated to muscle and plasma miRNA abundances. Am. J. Physiol. Endocrinol. Metab..

[44] Gallo S (2016). Stem Cell-Derived, microRNA-Carrying Extracellular Vesicles: A Novel Approach to Interfering with Mesangial Cell Collagen Production in a Hyperglycaemic Setting. PLoS One.

[45] Turchinovich A, Drapkina O, Tonevitsky A (2019). Transcriptome of Extracellular Vesicles: State-of-the-Art. Front. Immunol..

[46] Bell E, Taylor MA (2017). Functional Roles for Exosomal MicroRNAs in the Tumour Microenvironment. Comput. Struct. Biotechnol. J..

[47] Skog J (2008). Glioblastoma microvesicles transport RNA and proteins that promote tumour growth and provide diagnostic biomarkers. Nat. Cell Biol..

[48] Kemp Graham, Böning Dieter, Beneke Ralph, Maassen Norbert (2006). Explaining pH Change in Exercising Muscle: Lactic acid, Proton Consumption, and Buffering vs. Strong Ion Difference. American Journal of Physiology-Regulatory, Integrative and Comparative Physiology.

[49] Antoniou, A. *et al*. The dynamic recruitment of TRBP to neuronal membranes mediates dendritogenesis during development. *EMBO Rep* **19**, 10.15252/embr.201744853 (2018). 10.15252/embr.201744853 PMC5835843 29263199

[50] Lee DE (2016). microRNA-16 Is Downregulated During Insulin Resistance and Controls Skeletal Muscle Protein Accretion. J. Cell Biochem..

[51] Gao F (2017). miR-342-5p Regulates Neural Stem Cell Proliferation and Differentiation Downstream to Notch Signaling in Mice. Stem Cell Rep..

[52] Cheng S, Cui Y, Fan L, Mu X, Hua Y (2018). T2DM inhibition of endothelial miR-342-3p facilitates angiogenic dysfunction via repression of FGF11 signaling. Biochem. Biophys. Res. Commun..

[53] de Chevigny A (2012). miR-7a regulation of Pax6 controls spatial origin of forebrain dopaminergic neurons. Nat. Neurosci..

[54] Pinto SK (2017). Expression of microRNAs and target proteins in skeletal muscle of rats selectively bred for high and low running capacity. Am. J. Physiol. Endocrinol. Metab..

[55] He H (2018). MicroRNA expression profiles of neural stem cells following valproate inducement. J. Cell Biochem..

[56] Saika R (2017). MicroRNA-101a regulates microglial morphology and inflammation. J. Neuroinflammation.

[57] Miao B (2010). Small molecule inhibition of phosphatidylinositol-3,4,5-triphosphate (PIP3) binding to pleckstrin homology domains. Proc. Natl Acad. Sci. USA.

[58] Rafalski VA, Brunet A (2011). Energy metabolism in adult neural stem cell fate. Prog. Neurobiol..

[59] Hossain MS (2013). Plasmalogens rescue neuronal cell death through an activation of AKT and ERK survival signaling. PLoS One.

[60] Zhou H (2019). Angiopoietin-2 induces the neuronal differentiation of mouse embryonic NSCs via phosphatidylinositol 3 kinase-Akt pathway-mediated phosphorylation of mTOR. Am. J. Transl. Res..

